# Supplementary material for: Sorption of Sulfamethoxazole on Inorganic Acid Solution-Etched Biochar Derived from Alfalfa
Source: Materials (Basel). 2021 Feb 22;14(4):1033. doi: 10.3390/ma14041033 (PMC7926576; doi:10.3390/ma14041033)
Supplement: Supplementary file 1 [file materials-14-01033-s001.zip › materials-1090821-supplementary.pdf]

materials

MDPI

Supplementary Materials

Removal of Sulfamethoxazole on Alfalfa Biochar Etched with Inorganic acid Solution

Qi Li [1,*], Wei Yu[1], Linwen Guo[2], Yuhang Wang[1], Siyu Zhao[1], Li Zhou[1] and Xiaohui Jiang[1]

[1] College of Urban and Environmental Sciences, Northwest University, Xi'an, 710127, China; yuwei@stumail.nwu.edu.cn (W.Y.); yuhangwang895@stumail.nwu.edu.cn (Y.W.); 201931906@stumail.nwu.edu.cn (S.Z.); zhouli233@stumail.nwu.edu.cn (L.Z.); xhjiang@nwu.edu.cn (X.J.)
[2] Ningdong Forestry Bureau of Shaanxi Province; qili726@163.com
* Correspondence: qili726@nwu.edu.cn

Supplementary Materials and Method

Table S1. The adsorption kinetics parameters of SMX onto BCs.

Materials	Pseudo-first Order			Pseudo-second Order		
	K_1/min^{-1}	q_e/mg·g^{-1}	R^2	K_2/g·mg·min^{-1}	q_e/mg·g^{-1}	R^2
BC	1.8220	15.2260	0.7763	0.1483	16.1364	0.8878
ClBC	4.1179	26.6570	0.9615	0.2798	27.4057	0.9931
PBC	4.2531	26.7721	0.9613	0.2913	27.5099	0.9932

BC, ClBC and PBC were the biochars derived from alfalfa (AF) etched by H_2O, HCl and H_3PO_4

Table S2. Intraparticle diffusion model parameters of SMX onto BCs.

	K_{i1} /mg·g^{-1}·min$^{-0.5}$	C_1	R_1^2	K_{i2} /mg·g^{-1}·min$^{-0.5}$	C_2	R_2^2	K_{i3} /mg·g^{-1}·min$^{-0.5}$	C_3	R_3^2
BC	0.4822	6.7639	0.9824	0.2283	8.7434	0.9996	0.0559	14.1833	0.9757
ClBC	1.4502	12.8422	0.9316	0.1295	23.6407	0.8787	0.0237	26.5674	0.9642
PBC	1.4631	12.8910	0.9317	0.1387	23.6899	0.9223	0.0175	26.9628	0.8999

Table S3. The adsorption isotherm parameters of SMX onto BCs.

	T/K	Langmuir			Freundlich			Temkin		
		q_m	K_L	R^2	n	K_F	R^2	B	K_T	R^2
	288.15	25541.7397	0.0003	0.9961	3.0618	7.6514	0.9971	0.5288	4.6999	0.9618
**BC**	298.15	6288.2049	0.0013	0.9686	2.8576	7.8667	0.9765	0.5174	4.7201	0.9162
	308.15	10534.6537	0.0008	0.9543	2.6463	7.9989	0.9658	0.4901	4.4452	0.8874
	288.15	39.8651	1.3149	0.9914	3.5923	18.3598	0.9172	0.3769	27.7160	0.9881
**ClBC**	298.15	47.3483	1.1032	0.9960	3.9041	21.2197	0.9520	0.4294	70.0942	0.9984
	308.15	48.8470	1.4555	0.9911	4.3418	24.5997	0.9518	0.4719	197.6544	0.9981
	288.15	39.9958	1.3863	0.9795	3.5109	18.6072	0.9167	0.3665	26.8168	0.9831
**PBC**	298.15	47.6726	1.1976	0.9897	3.7836	21.9149	0.9479	0.4066	65.2313	0.9975
	308.15	51.6488	1.4481	0.9946	4.7250	26.4669	0.9554	0.5150	453.7562	0.9960

Table S4. The thermodynamic parameters of SMX onto BCs.

	T/K	ΔG/KJ·mol^{-1}	ΔH/KJ·mol^{-1}	ΔS/KJ·mol^{-1}·K^{-1}
	288.15	−18.1338		

BC	298.15	−18.8319	1.6351	0.0686
	308.15	−19.5062		
	288.15	−13.9147		
ClBC	298.15	−13.9625	3.9382	0.0613
	308.15	−15.1408		
	288.15	−14.0414		
PBC	298.15	−14.1660	1.7494	0.0543
	308.15	−15.1277		
